# In Vitro Evaluation of Nasal Aerosol Depositions: An Insight for Direct Nose to Brain Drug Delivery

**DOI:** 10.3390/pharmaceutics13071079

**Published:** 2021-07-14

**Authors:** Aida Maaz, Ian S. Blagbrough, Paul A. De Bank

**Affiliations:** Department of Pharmacy and Pharmacology, University of Bath, Bath BA2 7AY, UK; am3396@bath.ac.uk (A.M.); pd227@bath.ac.uk (P.A.D.B.)

**Keywords:** blood–brain barrier, drug delivery, nasal, nose to brain, olfactory, particle deposition

## Abstract

The nasal cavity is an attractive route for both local and systemic drug delivery and holds great potential for access to the brain via the olfactory region, an area where the blood–brain barrier (BBB) is effectively absent. However, the olfactory region is located at the roof of the nasal cavity and only represents ~5–7% of the epithelial surface area, presenting significant challenges for the deposition of drug molecules for nose to brain drug delivery (NTBDD). Aerosolized particles have the potential to be directed to the olfactory region, but their specific deposition within this area is confounded by a complex combination of factors, which include the properties of the formulation, the delivery device and how it is used, and differences in inter-patient physiology. In this review, an in-depth examination of these different factors is provided in relation to both in vitro and in vivo studies and how advances in the fabrication of nasal cast models and analysis of aerosol deposition can be utilized to predict in vivo outcomes more accurately. The challenges faced in assessing the nasal deposition of aerosolized particles within the paediatric population are specifically considered, representing an unmet need for nasal and NTBDD to treat CNS disorders.

## 1. Introduction

There has been some progress in drug delivery systems (DDS) for treatment for neurological disorders. However, effective drug delivery to brain tissues is still a great challenge due to complex anatomical and physiological barriers that selectively limit the entry of drugs into the brain. To work towards overcoming these challenges, recent advancements in the field of nanoparticle-based drug delivery have demonstrated promising results for nanotherapies in the possible treatment of CNS disorders [1]. Studies have revealed that nanocarriers enhance efficiency and are promising feasible formulations, making them an important target for research in both preclinical and clinical practice. However, the presence of the blood–brain barrier (BBB), which protects the brain from the entry of toxic substances, is a crucial obstacle for therapeutics to access the CNS. Therefore, novel brain targeting DDS are required to overcome this barrier and to enhance drug potential to treat CNS diseases [2,3]. The advantages of pulmonary DDS over oral delivery, such as rapid absorption and onset, enhance drug transport across the BBB when formulated in suitable sized and lipophilic aerosolized particles [4]. Promising outcomes for delivering CNS therapeutics via the lungs with the aid of different inhaler technologies have been reported for Parkinson’s disease [5], anxiety [6], analgesia [7], and migraine [8]. The olfactory region is the uppermost region in the nasal cavity exclusively connecting the external environment to the brain, unimpeded by the BBB. Over the last few decades, intranasal administration has emerged as an attractive non-invasive and direct route for the therapeutics to enter the CNS bypassing the BBB and avoiding hepatic metabolism and unacceptable systemic side-effects from other administration routes [9].

**Figure 1:**
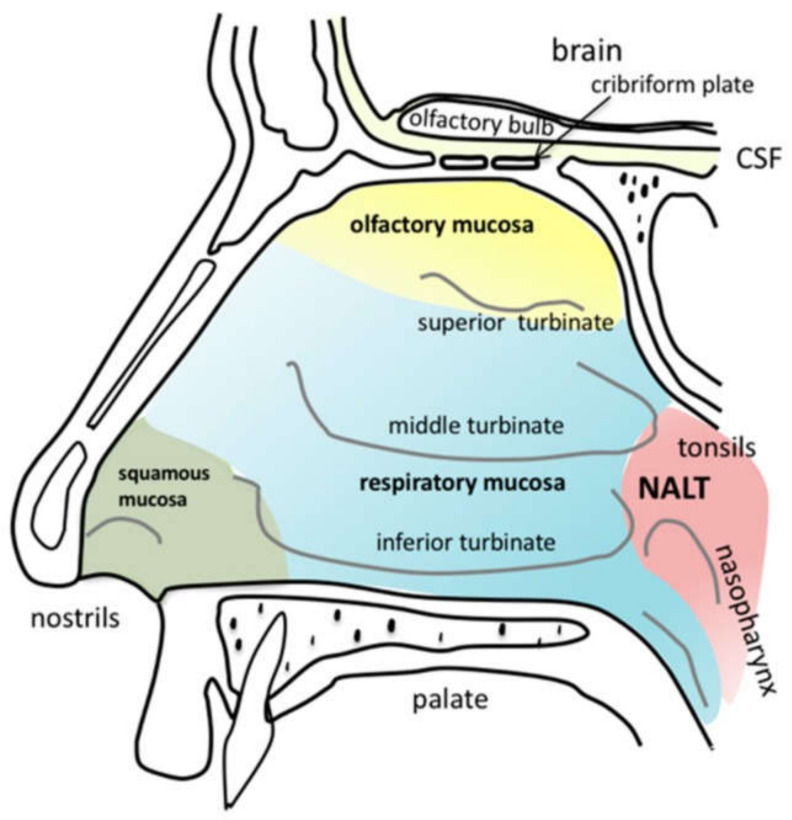
Anatomy of the human nasal cavity. Squamous mucosa (**green**) is located at the frontal parts of the nasal vestibules. The three turbinates (inferior, middle, and superior) humidify and warm the inhaled air. The area covered predominantly with respiratory mucosa is labeled in blue. The olfactory mucosa (**yellow**) is located next to the cribriform plate at the skull base down to the superior turbinate. Nasally transmitted substances can cross the cribriform plate via different pathways to enter the brain. Nasopharynx-associated lymphatic tissue (NALT) is located in close proximity to the tonsils at the nasopharynx. Reproduced from [13], MDPI, 2018.

In 1990, Frey II was the pioneer in proposing the nasal route for the delivery of neuro-therapeutic molecules, e.g., nerve growth factor (NGF) to the brain [10,11,12]. Research interest in nose to brain (NTB) drug delivery (NTBDD) systems continues to grow rapidly in an attempt to develop formulations that directly reach the brain at therapeutic concentrations [13,14,15]. Although the exact mechanism for NTB transport is not fully understood, three main pathways are involved: an epithelial pathway via the olfactory epithelium and neuronal pathways via olfactory and trigeminal nerves. The olfactory region is the uppermost region in the nasal cavity, which exclusively connects the external environment to the brain, unimpeded by the BBB (Figure 1) [13]. Drugs could then be transported via the olfactory epithelium intracellularly along the nerve axons or across the olfactory mucosa, transcellularly through the cells as well as paracellularly through the gaps between the cells [13]. In addition to delivery to the olfactory region of the brain, drugs can also find their way to the brainstem via the trigeminal nerve, which extensively innervates the respiratory mucosa in the nasal cavity. The branching of the trigeminal nerve makes it difficult to differentiate if the trigeminal pathway, olfactory pathways, or all are involved in NTBDD [13]. Significant efforts have been made in tailoring nasal formulations to enhance the efficiency of drug olfactory deposition, and the transportation in the olfactory region towards the brain is also being investigated by employing novel conjugates [16], semisolid formulations [17,18], particulate formulations [19,20,21,22], and lipid-based formulations [23,24].

Despite the wealth of studies that claim successful NTBDD, the majority are limited to preclinical stage research mainly because of the lack of adequate clinical evidence. Most of the available quantitative and qualitative data in the literature addressing the deposition profiles produced by conventional nasal formulations/devices reported limited drug delivery to the olfactory region—the target region for direct NTBDD [25,26]. Potential limitations may arise from the narrow and small duct olfactory cleft (1–2 mm width), which is hidden deeply in the nose above the main nasal airways, and thus only a small fraction of the air flow can pass through the olfactory region [27].

To develop a suitable NTBDD system, challenges in nasal drug delivery have to be addressed, some of which are formulation- and device-related, while others are physiological constraints that determine drug retention and permeation in the nasal cavity [28,29,30]. For instance, limited nasal residence time due to mucociliary clearance requires the use of non-harmful excipients in nasal formulations, e.g., adsorption enhancers and mucoadhesive agents [31]. Despite the relatively high surface area of the nasal cavity (150–200 cm^2^) [32], the absorption and subsequent physiological response of inhaled medicines is unpredictable due to the variety of epithelia and morphological characteristics in the human nose. As a result, there is significant variability in nasal spray device performance within the confined structure of the nasal cavity [33]. Moreover, considerable additional differences in sprayable droplet deposition occur in the nasal cavity due to interindividual anatomical and physiological variability in addition to the administration procedure adapted by the individual patient [33,34]. To predict nasal product bioavailability and bioequivalence, the United States Food and Drug Administration (FDA) recommends that the physical characterization of the device-released aerosols, e.g., droplet size distribution, viscosity, and plume geometry, should be well determined [35]. However, while considering spray properties alone is useful to differentiate between various nasal devices, this alone cannot precisely predict in vitro regional deposition of aerosols in the nasal cavity and further translation to in vivo measurements. Nasal aerosol deposition profiles are critical for the clinical success of nasal drug delivery. In fact, deposition patterns are of great importance not only in the process of developing efficient nasal devices and pharmaceutical formulations, but also to optimize delivery protocols and administration instructions for more targeted delivery to specific regions, e.g., the olfactory region in the nasal cavity, while achieving less off-target drug loss.

As it is unlikely that nasal product developers will be able to carry out encyclopaedic testing in human volunteers and patients, in vivo models such as mice, rats, and monkeys have been generally used as surrogates for humans in order to evaluate the distribution of airborne materials within the nasal cavity and to provide a proof of concept for olfactory targeting and efficient brain delivery via pharmacokinetic and pharmacodynamic studies [16,17,19,20,36]. However, translating the results from these models to humans is largely debatable, not only because of the anatomical and physiological variations but also because different species have discrete inhalation patterns [37,38,39].

The use of cell culture models is a common in vitro approach in the study of nasal and NTB drug delivery. These include primary cells such as reconstructed human nasal epithelium [40,41], porcine respiratory and olfactory cells [42], or cell lines such as RPMI 2650 [43,44] and Calu-3 [45]. Cell models have been utilized to provide information on drug absorption and permeability, therefore predicting in vivo bioavailability of nasally applied formulations. The human olfactory mucosa is also under development to reflect the neuronal transport from the mucosa and to simulate the NTB pathway [46]. Important parameters should be considered while validating in vitro models for NTBDD. Primary cell lines have a short lifespan even with proper nutrient supplementation, whereas tumor cells are immortal, but lack some nasal epithelium function, e.g., mucociliary clearance, as they are unable to differentiate into ciliated or mucus-producing cells, which significantly affect drug transport through the epithelium [42]. As an alternative to cell cultures, ex vivo models have also been used in NTBDD studies. Intact nasal tissues were isolated from animal or human donors and used for detailed examination of time-related transportation of nasal formulations across the tissue with control over physiological conditions, e.g., temperature, pH, glucose, and oxygen concentrations [47,48,49]. NTBDD is a strategy to bypass the BBB whereby therapeutic antibodies can be used to treat neurological diseases. The neonatal Fc receptor (FcRn) plays an important role in the transepithelial transcytosis of immunoglobulin G (IgG). The functional FcRn is in nasal respiratory mucosa; evaluating its role in drug delivery was determined in ex vivo porcine olfactory mucosa [50].

Although experimental validation is essential to predict in vivo aerosol distribution patterns, a possible means of overcoming the shortage of human participants in NTBDD studies and limitations of animal models is the use of computational fluid-particle dynamics (CFPD). Simulations using CFPD are a well-established research tool in exploring total and sub-zonal dosimetry of sprayed particles in the nasal cavity [51,52]. The numerical meshes, which originate from realistic in silico nasal airways, offer useful deposition information over a wide range of parameters, i.e., particle size distribution, velocity, and aerosol pattern, and they are considered a simple and relatively time-saving methodology in comparison to in vitro/in vivo validation [51,52].

A physical alternative to CFPD is the use of nasal casts. The enormous development in 3D printing technologies such as stereolithography (SLA), fused deposition modeling (FDM), and selective laser sintering (SLS) and their promising applications in the healthcare sector [53], alongside the advances in qualitative and quantitative analytical methods, have enabled building explicit nasal replicas as practical and efficient tools for in vitro evaluation of nasal formulations and delivery devices, and they could provide valid preliminary data for clinical trials [54]. Three dimensional nasal replicas were first described in 1978 [55], successfully derived from computer tomography (CT) scans and magnetic resonance imaging (MRI) and widely utilized to reflect the anatomical morphologies of the human nasal cavity and to measure the deposited nasal doses in vitro.

Nasal casts offer the advantages of resolving deposition partitioning in the nasal cavity yet avoiding exhaustive testing in humans and animals. Simultaneously, this approach enables nasal product manufacturers to understand thoroughly the elements that influence their product quality, and therefore they can define the limitations associated with safety and/or efficacy quality aspects of the product. Moreover, it has been suggested that it is possible to enhance the bio-relevance of the fabricated nasal replicas by using printing materials that imitate physiological conditions, e.g., being soft and stretchable with an adjustable nasal valve. Other approaches include coating the inner surface of the cast with artificial nasal mucus or introducing cells to manifest the characteristics of nasal epithelia [28].

Studies that have investigated in vitro aerosol deposition using nasal replicas have some limitations. The majority of them have dealt with a single nasal morphology model of an adult individual. Obviously, there are intersubject variabilities according to their age [56], gender [57], and ethnicity [58]. Therefore, it is speculative that a single nose morphology model will generally demonstrate approved regional targeting within the nasal cavity among widely different populations. Moreover, despite the improved methods for alignment and segmentation of different nasal regions in the model cast, there is a lack of standards that deposition studies can follow, e.g., the vertical line that splits anterior-posterior regions in the nasal cavity is still problematic where minor variations in replica cutting planes may ultimately lead to major shifts in regional aerosol nasal distribution. Table 1 shows the advantages and shortcomings of the preclinical models mentioned above, which are commonly used to evaluate nasal formulations and devices.

This review comprehensively and critically covers in vitro evaluation of nasal formulation deposition by utilizing human nasal replicas for a better understanding of the correlation between the various interacting factors with respect to the formulation’s physical properties, the device used, the validity of the nasal cast, and qualitative and/or quantitative analysis methods. A deeper insight is being gained into the posterior deposition, where the olfactory region is located in the nasal cavity and which is being thoroughly investigated as an interesting target area for direct NTBDD.

## 2. Nasal-Formulation-Related Factors

### 2.1. Aerosol Droplet Size Distribution

Whereas some researchers reported a minimum influence of droplet size on deposition efficiencies [63], others pointed out that droplet size is an important factor in the deposition pattern, where the larger it is, the less penetration beyond the anterior region will be obtained and the more concentrated droplets in the nostrils and at the nasal valve will be [64]. As for dry powder formulations, the ideal particle size suitable for nasal delivery is on the microscale (<10 µm) [54,65]. However, particles below this range tend to agglomerate spontaneously due to their high surface-to-volume ratio and subsequent cohesive forces, which makes the final acquired nasal localization is governed by the auxiliary airflow along with the capability of device to segregate particle mass [66].

Wingrove et al. carried out an in vitro comparison of the spray characteristics of two simple commercial nasal spray pumps with three different actuator models for their best upper nasal cast deposition [67]. Of these devices, the design that produced droplet sizes considerably larger than 50 µm favored anterior deposition (mainly at the nasal vestibule), and particles were unable to flow freely within the nasal perplexed anatomy. Schroeter et al.’s numerical model showed that within the 2.6–14.3 µm particle size range, particles of 10–11 µm diameter achieved maximum deposition in the central nasal areas. The fabricated replica cast was sectioned into six regions, within which particle distributions were 15% at the anterior turbinates—the region that confines the anterior segments of the middle and inferior turbinate, 7% at the olfactory region, and 12% at the turbinates, which include the majority of the middle and inferior turbinates posterior to the nasal valve. These values were in a good agreement with the simultaneous experimental measurements conducted in a replica cast [68]. The authors explained that the significant discrepancy in their results from previous studies was due to the variation in their anatomical definitions. For instance, in the studies of Shanley et al. [69] and Ghalati et al. [70], the nasal valve region covered a considerably larger surface area and thus much higher deposition estimation (~50% and 64% respectively) than the 29% deposition at the nasal valve reported by Schroeter et al. [64]. Similarly, the three-sectioned nasal cast in Liu et al.’s study [71] showed maximal deposition for 8 µm particles in the main middle region, which could not be further subdivided into anterior turbinates, turbinates, and olfactory regions clearly defined in Schroeter et al.’s nasal model. Swift et al. performed nasal deposition studies for nanoparticles over the volumetric flow rate from 1.4 to 50 L/min [72]. Four different nasal replicas at different research institutions were tested. The authors expressed one equation to estimate the diffusional nasal deposition of particle mean sizes between 4.6–200 nm, where the ultrafine particle depositions were mainly associated with particle diffusion coefficient and the applied flow rate with no significant correlation with age or nasal passage dimensions [72].

Xu et al. studied the feasibility of the nasally administered OVA/AS04 vaccine to induce local and systemic immunity in a mouse model, supported by in vitro vaccine deposition efficiency in the posterior nasal cavity, where the immune response is mainly produced [73]. In their study, the posterior section of the 7-year-old subject nasal cast represents the three turbinate regions. Using an Intranasal Mucosal Atomization Device (MAD Nasal™), the OVA/AS04 vaccine with droplet diameters with median volume distribution (Dv_50_) of ~266 ± 42 µm achieved more than 40% posterior deposition compared to 25% for the control vaccine formulation OVA/MPL, which was mainly recovered in the nasopharynx region (~50%) and was more likely to travel down to the lung due to its smaller droplet size of 132 ± 36 µm [74]. Table 2 shows the reported regional deposition of some nasal formulations in comparison to their particle/droplet size distribution.

### 2.2. Formulation Viscosity

Rheological properties that influence spray droplet sizes have a great impact on spraying behavior and deposition pattern. Nižić et al. used a combination of pectin, gellan gum, and sodium hyaluronate as a polymeric in situ gelling system to develop a sprayable fluticasone nasal suspension with an increased turbinate deposition posterior to the nasal valve as a targeted area for the treatment of nasal polyps. The chosen high-turbinate deposited formulations showed enhanced viscosity upon gelation. However, even high-viscosity formulations corresponded to a general decrease in turbinate deposition when the inspiratory air flow was reduced (the studied range was 0–60 L/min), as it caused a wider cone angle [74]. Moraga-Espinoza et al. achieved 84% turbinate deposition when the highest viscosity formulation containing the highest hydroxypropyl methylcellulose (HPMC, hypromellose) concentration (0.4%) was applied, giving a viscosity of 6.6 cP [59]. However, they pointed out that using viscosity as a factor to improve distal nasal delivery could be at the expense of patient comfort where the aerosolization efficiency will decrease and a jet-like spray will be formed [75]. Kundoor and Dalby’s study showed a remarkable decrease in the spray deposition region of nasal spray with increased viscosity and droplet sizes [54]. A Dv_50_ of 207 ± 5.4 µm and focused plume were obtained with the highest viscosity formulations (1565 ± 116 cP at 1 rpm), leading to a higher anterior deposition. Guo et al. showed that compared to high viscosity formulation (2% *v/w* methylcellulose, 18.2 ± 0.9 cP), the low viscosity formulation (0.25% *v/w* Avicel CL611, a microcrystalline cellulose (MCC), 4.0 ± 0.2 cP) provided wider surface coverage with higher middle and posterior deposition at 0 and 20 L/min and a slow inhalation pattern [76]. Similarly, formulations with lower viscosity produced smaller droplets (Dv_50_ = 37.7 ± 0.3 µm), which were swept by the air stream deeper into the nose, in contrast to bigger aerosol droplets (Dv_50_ = 199 ± 9 µm) created with high viscosity formulation, which were resistant to direction change due to inertial impaction at the front of the nasal cavity [76]. The deposition profile of low viscosity formulations, which is maximized in the middle nose where the respiratory region rich in blood flow is located, could lead to an increase in systemic drug delivery.

Pu et al. studied in vitro spray deposition of nasal solution formulations with different viscosities by adding Avicel or HPMC as a viscosity enhancer [77]. Interestingly, the authors found that spray properties and subsequent deposition were not only influenced by the overall formulation viscosity, but also by the type of viscosity enhancer used, with HPMC having a much greater effect, although Avicel produced a higher formulation viscosity. At higher concentrations, both excipients reduced formulation anterior deposition and frontal drainage as straightforward results of increased formulation viscosity and droplet sizes. However, Avicel- and HPMC-containing formulations showed significant differences. While Avicel eliminated ‘forward’ dripping (down the nostrils) without changing the total deposition footprint, HPMC substantially altered the deposition towards the nasopharynx with clear ‘backward’ drainage, mainly because of its moderate viscosity-enhancing effect [77].

### 2.3. Dry Powder Aerosolization Properties

One of the main challenges of any inhaled drug delivery system, including nasal products, is to produce particle sizes that are suitable for aerosolization. This is especially true for dry powder formulations, where particles are prone to aggregate lowering the fraction that is inhalable. Loading/mixing the therapeutic particles with non-active ingredients having favorable physiochemical properties is a common approach to enhancing particle flowability. Selecting proper excipients is critical for formulators as the lowest possible amount is required due to safety concerns and considering that the human nose cannot accommodate more than 10–25 mg of powder per nostril per shot [78]. Lactose, a disaccharide and FDA-approved excipient for inhalation, is an efficient carrier for dry powder formulations that can improve aerosolization and deposition performance [79]. As a hydrophilic excipient, lactose eases powder wetting with the mucosal aqueous fluid and thus enhances drug release and the dissolution profile. Moreover, it can also influence formulation mucoadhesion and the transport of poorly soluble drugs across the nasal barrier [78]. Nižić et al. reported a significant improvement of total turbinate and olfactory region deposition (from 13.5% up to 40%) of nasal pectin/HPMC microspheres blended with lactose monohydrate 1:9 *w/w* [60]. Human serum albumin (HSA) is another endogenous material used to improve physical characteristics and aerosolization properties for powder formulations [79] and is repeatedly reported as an efficient particulate system for NTBDD [80,81]. Moreover, in vivo studies showed that both epithelial and neuronal transport and overall brain targeting efficacy were enhanced when albumin nanoparticles were applied nasally [82]. Kaye et al. developed microparticulate dry powder nasal formulations of human antibodies (IgG) to enhance their efficiency against airborne infections [66]. A combination of albumin, sodium chloride, and disaccharides as excipients was used to produce aerosolized spray-dried IgG microparticle standard formulations. While these deposited mostly at the nasal vestibule (~55%), adding leucine 1% *w/w* and Aerosil^®^ 10% *w/w* as additional excipients further improved the nasal “bioavailable” deposition (~45% of total delivered dose) on the turbinates, olfactory region, and nasopharynx. These results could be attributed to the enhanced microparticle aerosolization properties of leucine- and Aerosil^®^-containing formulations, mainly by reducing inter-particulate forces.

## 3. Device-Related Factors

### 3.1. Device System

**Figure 2:**
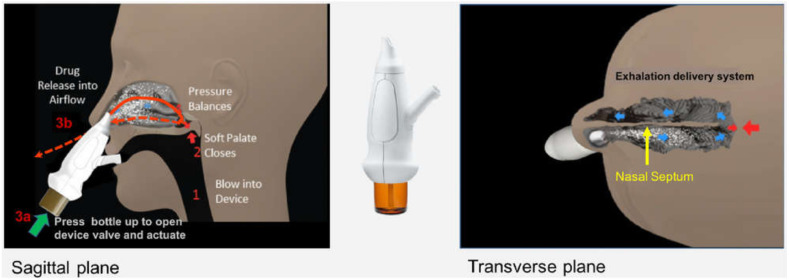
EDS mechanism. The EDS has a flexible mouthpiece and a nosepiece. The sealing nosepiece is shaped to transfer pressure from the mouth, to avoid compression of soft tissue in a way that could obstruct air flow, and to “stent” the nasal valve, particularly superiorly. Exhalation through the EDS (1) creates an airtight seal of the soft palate, isolating the nose from the mouth and lungs; (2) transfers proportional air pressure into the nose; and (3) helps “float” medication around obstructions to high/deep sites in the nasal labyrinth, such as the OMC. The transferred intranasal pressure is proportional, across various exhalation forces, to oral pressure, counterbalancing pressure on the soft palate. This assures a patient communication behind the nasal septum and allows air to escape through the opposite nostril. “Positive-pressure” expands passages narrowed by inflammation (vs. negative pressure delivery, “sniffing”). Use is simple and quick. A patient inserts the nosepiece into one nostril and starts blowing through the mouthpiece. This elevates and seals the soft palate, as with inflating a balloon, separating the oral and nasal cavities. The patient completes use by pressing the bottle to actuate. This causes a coordination-reducing valve to release the exhaled breath concurrently with aerosol spray in a “burst” of naturally humidified air. Reproduced with permission from [84], John Wiley & Sons, Inc., 2018.

It was reported that the mechanism by which a nasal device delivers its cargo has an immense impact on cargo destination within the nasal cavity. Djupesland et al. were interested in the postsurgical liquid deposition of different nasal device systems in chronic rhinosinusitis patients [83]. Three devices containing liquid medications were tested: high-volume low-flow (HVLF) nasal irrigation, conventional low-volume nasal spray, and a breath-powered nasal device (exhalation delivery system, EDS) (Figure 2); EDS is explained in detail in the EXHANCE-12 clinical trial [84]. The delivery efficiencies into sinus cavities of the three studied devices were assessed using a silicon cast produced from a postsurgical 47-year-old chronic rhinosinusitis patient (Draf II and Draf III procedures). HVLF and EDS mechanisms induced deeper penetration throughout the anatomically corrected sinuses, including the posterior/superior region in the nasal cavity. Both systems were superior to conventional spray, which produced limited liquid distribution within the anterior nasal section. HVLF showed deposition differences with head tilting and anatomy between Draf II and Draf III. Similar distribution patterns were observed with EDS, which may also be considered a more efficient and user-oriented device for nasal drug delivery.

Xi et al. experimentally and numerically quantified and compared nasal deposition profiles of jet and vibrating mesh nebulizers with two different breathing patterns: normal and bidirectional [85]. The bidirectional nasal delivery technique was originally invented by Djupesland et al. [86] and limits lower airway deposition following nasal administration. The technique makes use of the fact that oral exhalation naturally uplifts the soft palate closing the nasopharynx. Blowing into the delivery device allows formulation release into an airstream to enter the nasal passage from one nostril, and as the posterior connection with the mouth is blocked by the soft palate, the formulation will travel through the other nasal passage and eventually come out from the other nostril. The bidirectional technique holds many advantages for nasally applied formulations: it increases formulation residence time in the nasal cavity, the resulting positive pressure could enlarge the narrow compartments (nasal valve) in the nose, and, most importantly, it suppresses lung formulation deposition [86]. Xi et al. confirmed that for both tested nebulizers, the bidirectional breathing pattern was superior to standard nasal delivery in improving both overall and olfactory deposition. However, the advantage of this approach was much more prominent when a vibrating mesh nebulizer was used, where a 2.2-fold deposition increase was achieved in the nasal cavity and a 3.3-fold increase was achieved in the olfactory region [85]. As expected, different deposition patterns were observed in the two nasal passages using the bidirectional delivery technique, where much less of the aerosol dose was deposited in the second (out) passage. The same research group also compared four commercially available nasal spray pumps with four other nebulizers with different aerosol creating mechanisms: vibrating mesh, ultrasonic waves, jet with pulsating flow, and high-speed jet [87]. The point-release administration technique for the nebulizers was also tested, where four points in the nostril were selected to release the aerosol rather than the entire nostril. The deposition of nasal spray was mainly in the anterior region and, as such, the pumps are not suitable devices for olfactory targeting. Nebulizers, on the other hand, achieved considerable deposition beyond the nasal valve, and the best performance with the highest aerosol dose in the upper nose (8%) was observed for the mesh nebulizer. Although the point-release protocol applied resulted in 9.0 ± 1.7% aerosol deposition fraction in the olfactory region, for all nebulizers, a negligible dose was measured at the olfactory region. Kundoor and Dalby also showed that nebulizers resulted in significantly greater deposition beyond the anterior region when compared to a nasal spray pump [61]. Similarly, the authors explained their findings with nebulizers as they create smaller droplet size, with low exit speed, and a boost of airflow generated from the compressed air was used to create the nebulized aerosol [61].

Djupesland et al. clinically compared their bidirectional OptiNose powder device with a conventional liquid aerosol delivered with a spray pump [88]. The Opt-Powder device showed higher upper posterior formulation accumulation of 18.3% ± 11.5 compared to 2.4% ± 1.8 for the spray, in which the deposition in lower regions was three-fold higher than with the Opt-Powder [88]. Another other clinical proof of concept study demonstrated that the bidirectional nasal delivery device precluded lung contamination with zero or minimal deposition observed (0.8 ± 2.0%; range −4.1–5.6%) in 16 participants in comparison to (22 ± 8%; range 12.2–39.3%) with a commonly used nebulizer [86]. Suman et al. conducted a clinical study to investigate nasal deposition patterns using a conventional aqueous aerosol spray pump and a modified nasal nebulizer [89]. The authors suggested that nasal delivery systems should create small and slow movable particles to cover a larger surface area. Nebulizers have the potential to fulfil these requirements as they result in increased deposition in the superior and posterior regions and a greater coverage area than a spray pump. This is more likely to be due to the smaller droplets produced by nebulizers (2–10 µm) but, nevertheless, a significant fraction (33.3% and 58% in two participants) of the nebulized aerosol found its way down into the lung [89].

Four different nasal spray pumps, VP-7, PF-35, PF-60, and PF-80, were tested in a study by Cheng et al. [64]. The depositions of the tested spray pumps were primarily in the anterior and turbinate regions, and differences between devices were associated with droplet distribution size as well as spray angles, which might be controlled by the spray pump design. Suman et al. compared in vitro tests to in vivo performance in human volunteers for two different spray pumps regarding formulation deposition and its pharmacokinetic and pharmacodynamic activity [33]. Although remarkable differences were found in the performance between the two tested devices in terms of droplet diameters and spray angle and pattern, no significant variations were observed in the nasal deposition profile between the two, where the *p* value was 0.4 and 0.32 for inner:outer and upper:lower regional deposition ratios, respectively. The authors proposed that a major fraction of the spray impacts the nasal cavity near the release point, notwithstanding the device’s in vitro performance. As such, in vitro tests of nasal spray pumps with relatively similar droplet sizes might be clinically irrelevant in terms of deposition and response. This is mainly because nose anatomy, administration technique, and the degree of variability in physiological parameters are dominant factors. The authors also stressed that well-validated nasal casts and/or computer models are needed for acceptable in vitro–in vivo correlation of deposition [33].

The outlet design of the nasal device has also shown a considerable effect on aerosol deposition. Hosseini and Golshahi demonstrated an enhanced inhaled dose by using a modified nasal adapter characterized by a narrow tip [90]. Similar findings were reported by Dong et al. [91] who investigated a nebulization device attached to three different inhalation units: a mask, a single-headed nozzle, and a double-headed nozzle. They delivered methylene blue solution to 3D-printed nasal models of paediatric and adult nasal cavities and also an adult postsurgical sinonasal tract. The single-headed nozzle had the smallest outlet size and therefore the highest aerosolization velocity, which eventually led to deeper droplet travel into the nasal passage and more effective sinonasal deposition compared to the other two inhalation units [91]. Table 3 shows qualitative deposition efficiencies for commonly used nasal drug delivery devices [92].

### 3.2. Droplet Velocities

It was presumed that high initial droplet speed favors deposition at the anterior region of the nasal cavity, especially as large particles with high velocity are more likely to experience anterior inertial impaction due to the sudden airstream direction changes at the nasal valve [93]. In comparison to spray pumps, small and slow droplets generated by nebulizers attained more superior and posterior deposition in the nasal cavity [89]. Likewise, Sosnowski et al. stated that high particle momentum results in a remarkable inertia-driven localization not too far in from the nostril and is not expected to achieve deep penetration along the narrow airducts in the nasal cavity [62].

### 3.3. Spray Geometry

Plume spray pattern and plume geometry, which outline the shape of the extending aerosol mist following actuation, are FDA recommended in vitro tests for the bioavailability and bioequivalence of nasally administrated products [94]. Indeed, plume characteristics have some important influence on drug deposition in the nasal cavity and are equally dominated by the nasal device and the formulation. Aerosol plume geometry could be evaluated indirectly by applying high-speed imaging techniques with or without laser illumination, which gives the advantages of focusing on a single plane at the plume centerline aligned with the laser sheet and capturing images free of blur due to motion. However, high-speed laser imaging cannot provide data about the plume electrostatic profile, which ultimately influences aerosol performance [95]. The captured images could then be analyzed with imaging processing software for reproducible spray plume geometry outputs.

**Figure 3:**
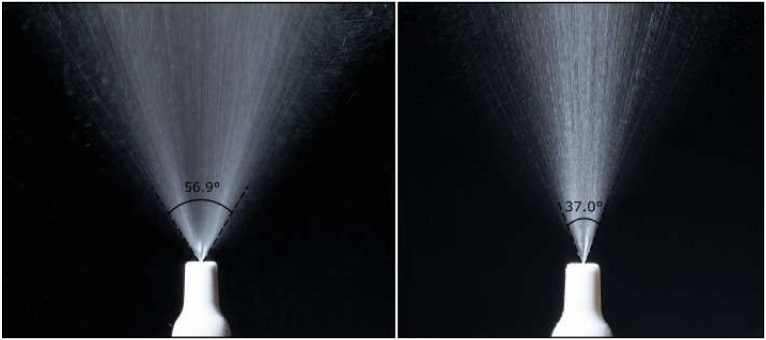
Spray cone angles of in situ gelling fluticasone (0.05%, *w/w*) suspensions prepared with (**left**) pectin (0.5%, *w/w*) and (**right**) pectin and gellan gum (0.5% and 0.2%, *w/w*, respectively). Reproduced with permission from [74], Elsevier, 2019.

Sosnowski et al. reported that wide aerosol cones were not compatible with the nostril geometry and that only the central part of the jet was able to penetrate deeply in the cast model [62]. Wingrove et al. described the pattern of plume jets narrower than 30° with relatively large droplet size as a duct rather than a mist, which is not ideal for upper superior region deposition [67]. The selected plume angles in this Wingrove et al. study for subsequent NTB insulin delivery in humans were in the range 30–45° in agreement with [74]. Adding gellan gum to make an in situ gelling fluticasone suspension led to a narrower plume angle (Figure 3) and thus higher turbinate deposition [74]. On the other hand, Hosseini et al. showed similarities in deposition patterns between two commercial metered dose nasal sprays when they were applied on the same nasal replica, with about 60% deposition in the anterior region. Both devices created different droplet sizes and plume angles: Dν_50_ 57 ± 1.25 µm, 35°, for Flonase^®^ Sensimist™ and Dν_50_ 126 ± 3 µm, 20°, for Flonase^®^. As the former has smaller droplet sizes but a wider plume angle than the latter, these two crucial factors might have neutralized each other’s outcomes [96].

**Figure 4:**
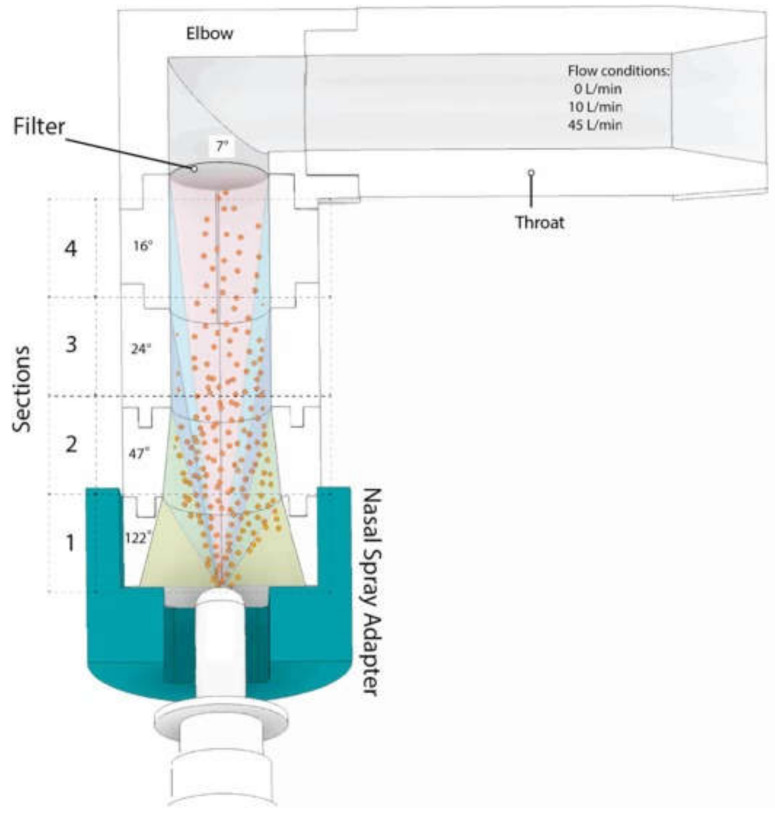
Experimental layout for use of the Plume Induction Port Evaluator (PIPE) with nasal sprays. The illustration represents in colors the effective angles used to calculate mass median plume angles (MMPA). Reproduced with permission from [75], Elsevier, 2018.

Moraga-Espinoza et al. used the Plume Induction Port Evaluator (PIPE) apparatus to evaluate the mass median plume angles (MMPAs) of four nasal formulations with increased viscosities (Figure 4) [75]. PIPE is an alternative protocol to laser imaging to characterize spray plumes by direct quantification of the deposited drug in the sectioned PIPE, which is caused by the direction of aerosolized droplets [97]. The results were in a similar trend to previous studies that used laser-assisted and high-speed imaging techniques where viscous formulations created narrower plume angles and higher turbinate deposition [75].

Sawant and Donovan also emphasized the importance of assessing the plume angles when developing nasal formulation/device composition and considered whether the anterior deposited dose, while using >26° plumes, was within the acceptable range [98]. Xi et al. compared different nasal devices along administration protocols for formulation distribution in the nasal cavity, and they were particularly interested in the olfactory region deposition [87]. Although the four tested nasal sprays in Xi et al.’s study were mainly deposited in the anterior/nasal valve region, the spray with the narrowest plume 19 ± 0.6° could indeed infiltrate into the superior meatus, depositing a small fraction at the olfactory region, ~4.6% of the total nose dose [87]. A comparable trend was also observed in the Foo et al. study, which provided a proof of concept that spray plume angle alone is a valid and accurate factor in predicting nasal deposition profile [63]. Almost 90% of deposition efficiencies were achieved at the turbinates for small plume angle sprays <30° when combined with 30° administration angle, whereas 55–65° plume angle sprays caused ~75% turbinate deposition when a similar administration angle was applied [63]. The authors stressed the fact that in order to obtain greater turbinate deposition with narrow plume geometries, the administration angle should direct the aerosol towards the nasal valve but not to walls, which otherwise requires plume angles wide enough to direct a sufficient fraction of the plume to the valve.

## 4. Patient-Related Factors

Device administration instructions for insertion into the nose have also been reported in some studies. Although Kundoor and Dalby acknowledge the significance of nozzle-tip depth and orientation effect on nasal droplet deposition [54], these two factors might not be of such great importance for children due to the relative large tip size in proportion to a child’s nostril, as demonstrated by Sosnowski et al. [62]. Sawant and Donovan reported essentially 100% anterior deposition in a nasal model from a 12-year-old subject. This effectively total loss was mainly due to the test setup and the use of stiff material for the cast anterior piece, hindering reasonable insertion of the spray nozzle into the nostril [98]. Similarly, in the study of Wilkins et al., a 16-month-old subject’s nasal model showed the lowest deposition efficiency in the nasal cavity and highest anterior loss compared to the other four studied infant models. This was potentially due to the low device insertion depth into the nostril as well as the longer anterior length of the 16-month-old subject [99]. On the other hand, Hosseini et al. used elastic material to build the anterior section of their studied casts to further simulate realistic conditions. Such flexibility allowed 3 and 5 mm proper device insertion through the nostril for the studied pediatric and adult nose models, respectively [96]. In their study, the anterior spray loss was ~60% for both the 2-year-old and 5-year-old subjects’ nasal casts when formulations with relatively similar size distribution to those of the Sawant and Donovan study were used.

**Figure 5:**
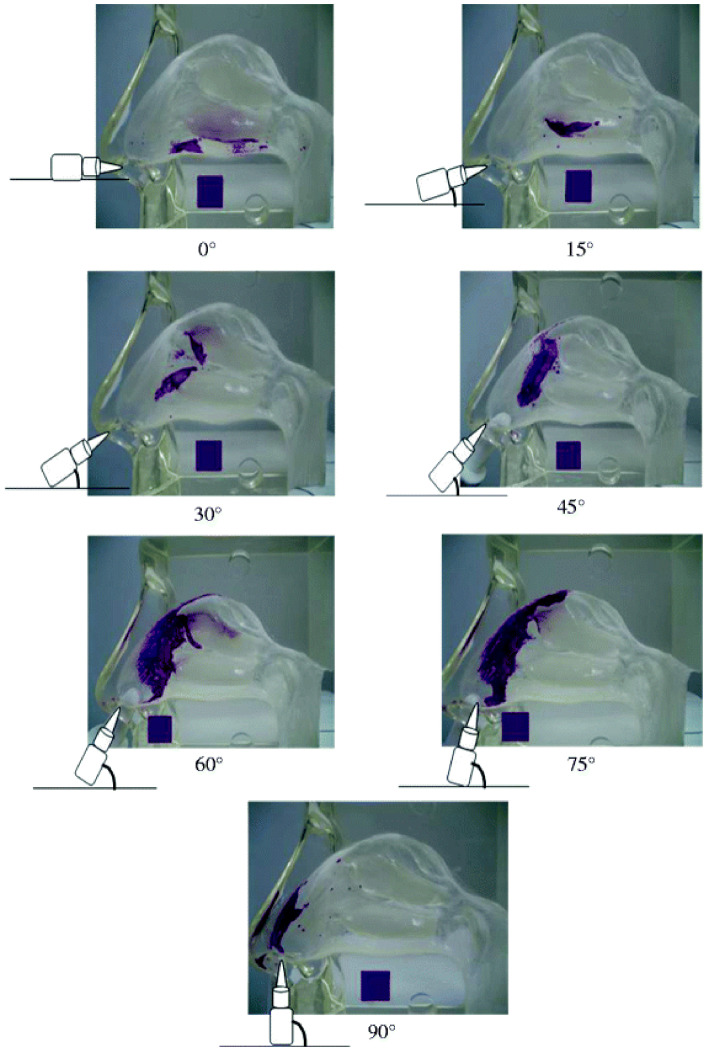
Deposition pattern of Afrin nasal spray at different administration angles (insertion depth = 5 mm). Reproduced with permission from [54], Springer Nature, 2011.

Likewise, the administration angle, which represents the angle between the nasal cast floor and the device tip, with the head perpendicular to the horizontal, seems to be a critical factor for efficient aerosol delivery in the nasal cavity. Foo et al., reported ~90% turbinate deposition efficiency at a 30° administration angle along with a narrow plume angle [63], whereas Kundoor and Dalby, who evaluated three marketed nasal sprays (Ayr, Afrin, and Zicam), showed that a reduced deposition area resulted from smaller administration angles, e.g., 0°, compared to larger ones (75°), with the greatest deposition in the nasal valve area achieved at angles ≥60° (Figure 5). The nasal spray was unable to expand within the constricted small area at small administration angles, leading to formulation accumulation [54]. Warnken et al. used patient-specific administration angles to evaluate turbinate deposition of Cromolyn sodium nasal solution in five pediatric-subject and five adult-subject nasal replicas. A general increase in turbinate deposition efficiencies with all casts was observed when the administration angle was decreased from 75° to 30°. However, the use of subject-specific administration angles improved the deposition to ~90% compared to 73% when the standard 30° angle was applied. Moreover, the advantages of subject specific administration parameters resulted in a decrease in deposition variabilities amongst the studied casts, which ranged between 75.7% and 97.8% compared to 46.4% and 87.9%, with the idealized administration angle of 30° [59].

Manniello et al. explored the variability in posterior nasal deposition amongst twenty nasal replicas from adult subjects (10 males and 10 females) using two locally acting nasal sprays, Flonase^®^ and Flonase^®^ Sensimist™ [100]. The study showed significant variability in deposition patterns associated with intersubject anatomical differences. The tested replicas exhibited a wide range of nasal posterior delivery in both sides, right (22–90%) and left (12–99%), when a 7.2 kg actuation force was applied. A smaller range of delivery variations was measured with Flonase^®^ Sensimist™ sprays than with Flonase^®^, which means that nose anatomical features could be less important for some nasal devices. The authors suggested that it is useful to report the posterior delivery range during nasal drug development and bioequivalence protocols of nasal products [100].

## 5. Other Factors

### 5.1. Airflow Rate

**Figure 6:**
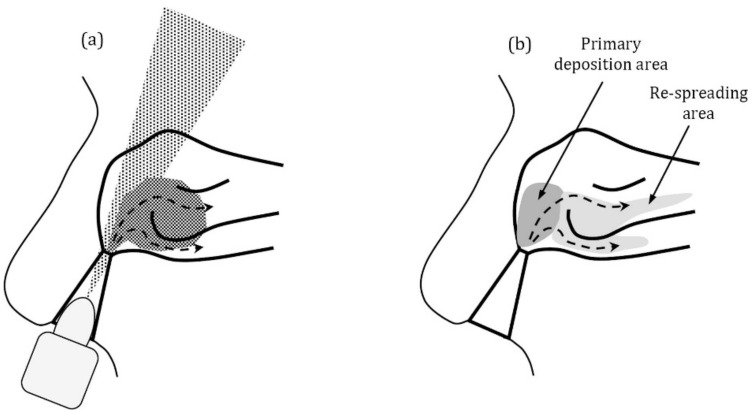
Proposed mechanisms of drug penetration during the simultaneous air inspiration: (**a**)—droplet entrainment by air during spray application (probably less important for large droplets), (**b**)—spreading of already deposited drug along the nasal surface due to the interactions with the air stream. Reproduced with permission from [62], Elsevier, 2020.

The association between the inspiratory airflow and aerosol deposition patterns in the nose is a debatable point. While some studies have concluded that there is no or only scant effect of the breathing profile on deposition [59,63,76], others showed deeper deposition beyond the nasal valve by increasing the flow rate [74,75]. Foo et al. confirmed that by tuning the spray plumes and administration angles, they could achieve mass deposition up to 90% in the three (physiological) turbinate regions, and increasing the inspiratory airflow to 60 L/min caused no change in turbinate localization [63]. However, Sosnowski et al. showed that adjunct airflow improved nasal drug transport by 2–3 cm towards the nasopharynx [62], but the authors considered that secondary spreading occurred as a result of active displacement of already-deposited drug layers covering the nasal surface was a more reasonable explanation for the enhanced drug transport, as illustrated in Figure 6. Such a phenomenon has great importance in the paediatric narrow nasal airways investigated by Sosnowski et al. [62]. Nižić et al. reported that the dry powder nasal deposition and recovery efficiency were decreased upon applying 20 L/min inspiratory airflow, which could be due to carrier/microsphere de-agglomeration associated with the assisting airflow, producing smaller particles that can easily leave the nasal cavity [60].

In the Moraga-Espinoza study, where the authors used a mass-based PIPE protocol to measure nasal spray plume angles, spray-cone angles narrowed with increasing viscosities, and this effect was more significant under hard-sniffing-like flow (45 mL/min) for all formulations except for the highest in viscosity using 0.4% HPMC. Their results showed that breathing conditions had an influence on the spray performance and caused distal drug distribution in the middle and lower turbinate areas, especially through a single open nostril [75]. They explained the argument around the airflow-deposition relationship in the literature by either utilizing low inspiration airflow or keeping both nostrils open, which limits the air velocity within the nasal cavity [75]. Xi et al.’s in vitro and numerical study revealed that higher inhalation flow rates reduced the total nasal surface coverage but enhanced the olfactory dose with both jet and vibrating mesh nebulizers [85]. The authors speculate on this phenomenon as follows: at a higher flow rate, gravitational sedimentation is less significant and particles become more inertial and more likely to escape the airstream. As such, particles will occupy the nasal floor if they enter from the lower nostril, as most of the particles do, and reduced total nasal deposition rate (vestibule and turbinates) will occur, whereas particles entering the nostril tip, with 60° from the horizontal plane, will deviate from the main airflow and keep their upward trajectory and most probably penetrate deeper towards the olfactory region when the inhalation flow rate is high [85]. Formulation with Avicel 0.25% at airflow 10 L/min achieved higher upper deposition compared to 20 L/min [76]. This could be attributed to the turbulence induced by higher airflow, which led to unpredictable directions. Moreover, the increased upper surface coverage at 10 L/min increases the chance of drug delivery to the olfactory region and possibly, therefore, to the CNS [76].

### 5.2. Cast-Related Factors

Kundoor and Dalby studied different head positions according to the label instructions of two marketed nasal products: Flonase (GSK) suggests tilting the head forward, whereas Nasacort (Rhone-Poulenc Rorer) promotes tilting the head backwards. While anterior deposition (at the nasal valve) was observed with forward tilting, the sprayed droplets reached deeper areas until the middle of the nose with the head tilted backwards [54]. High administration angles (≥60°) allowed spray droplets to expand better within the nasal cavity and to pass through the nasal valve. Their results agreed with those of other studies [59,63] on the importance of administration angle and hence head tilting for targeted deposition.

Doughty et al. were interested in some manually actuated nasal sprays’ variabilities, which include head position, breathing patterns, and hand techniques, and how together with formulation and device parameters, they can affect in vitro spray performance and, ultimately, the deposition in the nasal cavity [101]. Force- and velocity-controlled settings were investigated to mimic hand-actuating profile in adult and paediatric patients. Flonase nasal spray (Fluticasone propionate, GSK) was used in the study. The results showed that the reduction in actuation force and compression velocity during spraying, which are two paediatric related parameters, caused a significant decrease in spray weight, and the paediatric patients might receive only a partial dose or even no dose at all. Significant differences were observed in the spray droplet size distribution test, which was more sensitive than spray mass to actuation force, compression velocity, and force rise time variabilities [101].

Le Guellec et al. conducted a validation study of two nasal anatomical models obtained from a preserved cadaver head and its prototyped nasal cast from CT images by comparing the data with in vivo aerosol deposition in seven healthy volunteers [102]. Sonic jet and mesh nebulizers were used as delivery devices in the study, and the authors defined the deposition in three main regions, namely nasal, upper, and maxillary sinus. In contrast to the 3D-printed replica, which was constructed via fused deposition modeling, the plastinated-head model gave satisfactory predictions of regional deposition when both were compared to the in vivo scintigraphic measurements. Furthermore, although the two models had a similar anatomical background, different nasal deposition profiles were attained. The authors explained the differences being due to the method used to fabricate the models where the anatomical and geometrical features of the nasal airways in the plastinated model closely mimicked human nasal anatomy. The roughness of the prototyped cast inner face could have caused flow disturbances and is more likely to increase aerosol impaction and holding in some parts in the nose. Lastly, the materials used to build each model are not similar and might have affected the radioactivity counted. The plastinated-head model is made by replacing the water and fat in a cadaver with certain plastics. The radioactive attenuation pattern in the studied areas was more homogeneous in such a model than in a 3D-printed cast, which was made of plastic [102]. In a previous study, Kelly et al. investigated the deposition efficiency of nanoparticles in two replicas made from the same source but via different stereolithography procedures, which could lead to alterations in the surface smoothness [103]. The authors extended their study by comparing their results with other nasal models featuring different nasal airway geometries. Deposition efficiency was mainly associated with particle diffusivity, with minimal nasal deposition observed for particle ranging from 30 to 150 nm, suggesting particle filtration down to the lung, whereas increased deposition was noticed with increased diffusivity for particles smaller than 30 nm. Neither the processes applied to build the nasal models nor the small variations in the nasal airways between the models had a pronounced impact on nasal deposition of ultrafine particles (<150 nm) [103]. Conflicting findings were reported by the same research group and in a similar study on the nasal deposition of microparticles (1–10 µm), where the deposition patterns of inertial particles were sensitive to replica manufacturing methods, replicas surface roughness, and nasal geometry differences [104]. Zhou et al., found some differences between experimental and CFD data that could be attributed to the fact that the computer model surface is smoother than the nasal replica used with unavoidable artefacts and surface roughness due to the limitation of the applied prototyping techniques [105].

Djupesland et al. compared a Koken silicon cast (Koken Co.), which is often reported in the literature for in vitro evaluation of nasal deposition [61,76,77,84], with an Optinose nasal model, which is designed to mimic the oropharyngeal geometries while using the EDS system where the soft palate is elevated, sealing the velum that separates nasal and oral cavities [106]. Geometry and shape assessments showed that the Koken cast had a significantly larger nasal volume and minimal cross-sectional areas compared to the Optinose model, as well as the typical in vivo ranges for both genders. The authors questioned the validity of nasal replicas for accurate and definitive in vitro drug delivery studies, where such differences will ultimately influence the intranasal pressure and flow characteristics and therefore drug deposition patterns [106].

### 5.3. Airway Expansion

Expanded nasal geometries can enhance the ventilation of the upper nose and eventually elevate the olfactory region drug delivery, as was shown by Xi J. et al. [107]. They investigated deposition alterations relative to different nasal dilation degrees while using two nasal delivery methods: the conventional unilateral method and a novel bidirectional method invented by Djupesland et al. [86]. Although the overall delivered dosage to the nose was lower with airway dilation, reduced pressure and increased airflow fraction were produced at the superior meatus, which is normally under-ventilated in the nasal cavity. As a result, olfactory delivery was improved by 2.2-fold and 4-fold with unilateral and bidirectional protocols, respectively. Using numerical modeling, the authors noticed two distinctive flow partitions that could explain the enhanced olfactory localization in enlarged noses. The first was generating a low pressure-recirculation area in the mid-meatuses, which enhanced the upper nose ventilation and was noticed in both nasal pathways (left and right), whereas the second flow pattern was dense ventilation observed at the outlet nostril (second nasal pathway) when the bidirectional protocol was employed. Xi J. et al. concluded by suggesting active nasal dilution, e.g., using an expiratory flow resistor, nasal decongestants, and nasal dilation devices as an effective parameter to increase olfactory region targeting in the nasal cavity in line with nasal positive pressure using a bidirectional delivery approach.

## 6. Nasal Deposition Studies in Pediatrics

Little information is available about the performance of nasal devices and formulation deposition for paediatric subjects. There is an unmet need for nasal drug delivery assessment in the child population owing to the developing demand for nasal pharmaceutical products for paediatric patients. In fact, there are distinct child/adult inter-individual differences in the nasal morphologies as well as inhalation patterns [96]. Laube et al., were the early researchers who investigated the paediatric nasal deposition profile using a 9-month old Sophia Anatomical Infant Nose–Throat (SAINT) model [108]. Radioactive dry powder formulation was delivered via a Solovent dry powder inhaler equipped with a holding chamber (125 mL volume spacer) to avoid force inhalation, which is not practical with an infant or young child, especially with the tidal breathing conditions used in the study. Three different tidal volumes were investigated, 50, 100, and 200 mL, and regional deposition was demonstrated as a ratio between the inner zone (most posterior third) to the outer zone (most anterior third). Although the device achieved 90% delivery efficiency, a significant amount of powder was retained in the spacer for all three tidal volumes, and the deposition was low in both regions studied. The deposition trend was tidal volume-dependent with the highest at 200 mL. As such, a lower deposited powder dose would be expected in infants breathing 50 mL compared to children breathing 100 and 200 mL. Golshahi et al. characterized in vitro deposition of microparticles (0.5–5.3 µm) in 13 nasal airway casts (extended to the upper trachea) obtained from 4–14-year-old children in an attempt to find an association between nasal airway geometric features and child inhalation patterns [109]. Moreover, five adult replicas were chosen for comparison. Deposition in child nasal replicas was impaction dominated and increased with higher flow rates and greater particle sizes. In addition, the dimensional parameters, trans-nasal pressure drop, and the two dimensionless Stokes and Reynolds numbers considerably reduced inter-subject deposition variabilities in child nasal airways. The impaction parameter is a factor that describes nasal deposition predominantly by particle impactions for different sizes on different flow rates [110]. By comparing deposition over different age groups while maintaining a steady impaction parameter, child (13 subjects) and adult (5 subjects) data were relatively close, but lower than previously reported infant data (11 subjects, 3–18-months old) [109].

**Figure 7:**
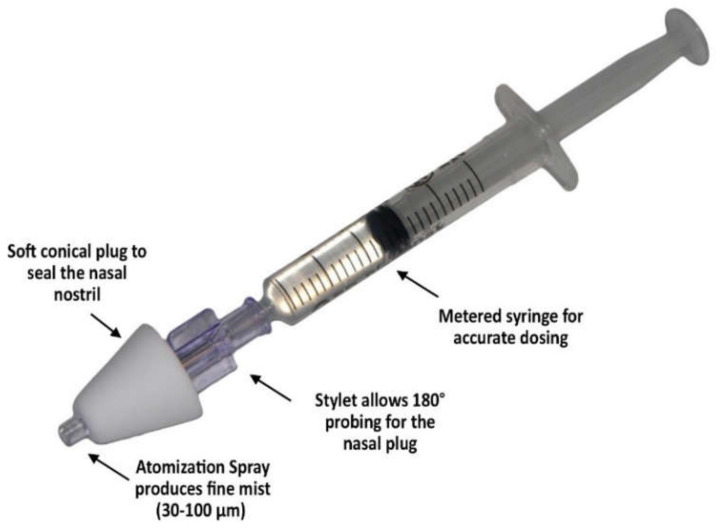
The Mucosal Atomizer Device (MAD) used to deliver medications via a fine spray in the nasal cavity. Reproduced from [111], Springer Nature, 2014.

Two marketed metered dose nasal sprays and a MAD Nasal™ (a syringe-based and hand-actuated device commonly used for the delivery of some CNS-therapeutics such as Midazolam, Fentanyl, and Ketamine to children via intranasal administration (Figure 7) [111]) were tested by Hosseini et al. for dose deposition in the three nasal models of a 2-year-old toddler, a 5-year-old child, and a 50-year-old adult [96]. In general, the two device types showed significant sub-regional nose deposition dissimilarities between adult and paediatric subjects. The nasal spray products caused higher formulation loss at the anterior region in child and toddler models (~60%) compared to adults (~40%). Olfactory deposition was not detectable in the two paediatric models, not reproducible (high RSD), and less than 2% in adults. For the MAD atomizer, the anterior filtration measured was ~10–15% in all three tested replicas. However, nearly 30% of the formulation that escaped down into the throat was detected. Additionally, no olfactory region deposition and meaningful variations in turbinate depositions were observed between the three subjects. The poor olfactory localization of both nasal sprays and the atomizer in all three replicas could be explained as being due to the large droplet size distribution (nearly 46–164 µm), lack of diffusion movement, and limited inspired air (~14%) passing through the olfactory region [112]. Surprisingly, the sub-regional deposition profile showed no correlated tendency with the nasal geometry of the three subjects. Turkey’s post hoc test revealed that the deposition differences were significantly and typically ascribed to the variations between paediatric and adult nasal replicas rather than the variations between the two tested nasal sprays or between the child and toddler nasal models [96].

The nasal valve is characterized by a minimal cross-sectional area (0.3–0.4 cm^2^), on each side, in the nasal cavity. It is a substantial barrier for the airstream to pass through and reach the main nasal cavity where the wide and highly vascularized nasal turbinate region is located. As children have a narrower nasal valve than adults, it will not be surprising to expect a greater anterior localization of nasal sprays in children compared to adults. Indeed, Sawant and Donovan detected no deposition beyond the nasal valve in a model made from a 12-year old for testing Valois VP-7 devices [113], and 100% of the drug was filtered by the anterior region, while 60–90% succeeded in entering the turbinate region in the nasal cast from a 53-year old when the same conditions were applied [98]. Although coating the inner surface of the cast with simulated mucus solution caused better spreading of the formulations throughout the cast, a slight improvement in the spray turbinate distribution was observed. These findings are most likely due to the child’s smaller nose geometry and narrower nasal valve. The authors explained the contradiction of their results compared with those of Laube et al. [114] as being due to various factors that seem to have a significant impact on the deposition in the turbinate region such as the child’s age, anatomical differences, the device tip insertion depth and angle into nose nostril, and the released plume angle. The Accuspray™ device used in the by Laube et al. was well-fitted into the nostril due to the flexibility of the nostril material and produced a narrower plume angle plume, allowing a greater jet fraction to enter the turbinate region beyond the nasal valve. Sosnowski et al. used a nasal cast from a 7-year-old with an elastic vestibule for a more realistic tip fitting in the nostril, and five nasal spray pumps with different atomizing nozzle shapes were tested [62]. The small dimensions of the child nose model along with the airflow allow the secondary mechanism of formulation transport deeper into the nose (an additional 2–3 cm). This phenomenon accounts for the liquid bridges created from deposited formulation layers that plug the very narrow airways in the paediatric nose. Such bridges could be breached by the airflow causing secondary aerosolization distally towards the nasopharynx [62].

Inter-subject nasal geometrical disparities remain a great challenge in aerosol deposition studies not only between adults and children but also within the same age group. For that reason, Javaheri et al. have developed an infant nose model that could be used as a simplified reference in aerosol delivery studies [115]. The idealized model has average geometrical features obtained from 10 previously presented nasal replicas of 3–18-month old infants [116], and the main aim of the developed model was to represent average nasal deposition and imitate realistic nasal passage. The reported deposition data from the 10 realistic infant nose models showed wide discrepancies due to geometrical variations. However, deposition in the idealized version displayed satisfactory agreement with the average of the 10 subjects considered in the Storey et al. study. The authors concluded their results by considering their model as an acceptable in vitro reference for aerosol distributions in infant airways [115]. Zhou et al. conducted computational calculations supported by in vitro tests for total and regional aerosol deposition in 5-year-old nasal airways [105]. Experimental and numerical data were relatively close in the overall deposition. Alongside particle size and flow rate, nasal geometry effects on deposition were evident in terms of surface area and pressure drop (impaction parameter), in agreement with Golshahi et al.’s results [109]. Regional deposition comparisons with an adult replica were also included in the study. Increased particle size enhanced the deposition in all regions. In particular, the anterior region received more particles in adult than in child replicas as it is doubled in length and more complicated morphologically in adults than in children [105]. Wilkins et al. have investigated regional vaccine nasal depositions in five nasal models of infant subjects aged 3, 5, 11, 16, and 24 months [99]. Despite inter-subject geometric variations, no significant differences were found in the total deposition efficiencies between the five models in general. Significant differences in the regional deposition efficiency were only observed in the anterior delivery in the 16 month model when compared to the 5-month-old and 24-month-old models [99].

## 7. Deposition Assessment Methods

Various qualitative and quantitative protocols have been investigated for spray deposition assessment following nasal administration to be a well-grounded analytical option for subsequent clinical studies [102,117,118]. Sartoretti et al. tested the validity of computed tomography (CT) imaging of iodinated nasal spray for in vitro deposition evaluation [119]. Unidose nasal device and a solution of 92.5 mg/mL of iodinated contrast agent (ICA) to be aerosolized within the nasal replica were used for the experiment. Unalloyed CT imaging utilizing ICA and a low-dose CT procedure (CTDI_vol_ 7.6 mGy) enabled rapid and precise visualization and localization of individual droplets before their gravitational agglomeration and overcame the drawbacks of other methods such as gamma-scintigraphy 2D-imaging where the image brightness is based on the particle concentrations. It is considered a slow imaging protocol, it lacks the hidden anatomical structure and/or overlapping surface observations, it could be influenced by camera direction/distance, and it still needs professional handling of the gamma-pharmaceuticals (radiolabeled aerosol with gamma-emitters such as technetium-99 m) [120]. Moreover, attenuation of gamma-rays could occur while penetrating body tissue, with about 50% of the released photons being scattered, causing poor measurement reproducibility [85]. However, Sartoretti et al. acknowledged some shortcomings of their developed CT-based imaging and its further application in deposition studies [119]. For instance, method parameters, e.g., slice thickness, have a great influence on the obtained image resolution and accuracy. The two tested software programs induced different volume recovery rates, and some of them were greater than 100%, which could be due to different thresholds and algorithms between the two software solutions. The considerable density differences between the nasal cast substance (or the air) and the iodine made even low iodinated voxels (3D pixels) show high Hounsfield unit values and high density, and therefore higher deposited droplets were assumed. Thus, Froehlich and co-workers [119] suggested further investigations into other software programs and nasal casts, considering the inspiration via the nose and using a technique that offers a greater distinction of materials that are required to determine accurately nasal aerosol deposition patterns.

3D single-photon emission computerized tomography (SPECT) is another imaging technique that requires radioactive aerosols and has been utilized to visualize particle depositions in the nasal cavity. SPECT-CT accurately associates 3D nuclear images to anatomical structures as used by Leclerc et al. to access the nasal and particularly sinusal deposition of micron (2.8 µm) and submicron (230 nm) radioactive aerosol [117]. 2D gamma-scintigraphy was also investigated, and both techniques were compared relative to radioactive counting as a reference. SPECT-CT approach proved to be efficient and was in good agreement with the radioactivity analysis, whereas the planar scintigraphy produced a nine-fold overestimation of maxillary sinuses deposition [117]. Despite SPECT providing valuable 3D aerosol spatial distribution details, especially when it is used with high-resolution computed tomography (HRCT), its cost, complexity, and the lack of relative anatomical distribution without HRCT are some of the shortcomings that cannot be ignored [120]. A quantitative temporal and spatial testing protocol for new radio-nanotherapeutic agents was also investigated in vivo. Intranasal aerosolized radiolabeled polymeric micellar nanoparticles were tracked with positron emission tomography/computer tomography (PET/CT) imaging in a rat model to measure NTBDD quantitatively [121].

The color-based method using moisture-sensitive Sar-Gel has been repeatedly reported in the literature as a valid qualitative procedure for in vitro nasal deposition and drug delivery studies [54,83,87]. Sar-Gel has a color-changing property from transparent to purple, which is highly sensitive to tiny amounts of water as low as 0.5 µL, and the fluctuating color depths, when digitally photographed, are proportional to the settled vapor mass from the released droplets, representing aerosol delivery. Control trials showed no noticeable variations occurred in Sar-Gel color on exposing the cast to ambient moisture within a 5 min period, which is sufficient to complete one test [54,61]. Xi et al. studied human mouth–lung replica deposition of three different nebulized aerosols [120]. Their qualitative results were in good agreement with computational modeling and in vitro experiments depending on Sar-Gel visualizing when the same conditions were applied. Although Sar-Gel imaging holds combined advantages with its straightforward application, sensitivity, simple processing, and relatively considered clinically acceptable imitation of inhaled drug delivery, it is limited to qualitative analysis. Several research groups have reported quantitative estimates using the color-based method. Pu et al. demonstrated the fractional nasal deposition as a percentage of overall deposition footprint using imaging software [77], whereas Nižić et al. and Xi et al. used an electronic scale for gravimetric analysis to determine the aerosol deposition rate as a weight difference between the empty nasal cast and following formulation administration to the delivery unit output [74,85]. Xi et al. reported 32 ± 4% underestimation of aerosol deposition using the colorimetry-based method compared to the mass weighing method, and they acknowledged the necessity of using the same materials and lightning conditions for satisfactory deposition-colorimetry correlation [87].

Aerosolized fluorescent particles passing through a six-section nasal cast were used in Schroeter et al.’s study [68]. The deposition was evaluated in each section by measuring the amount of deposited fluorescein using a typical 96-well microplate reader, and the sections were soaked beforehand in 0.1 M ammonium hydroxide to collect the particles. Swift et al. used four different nasal casts to investigate the deposition of radioactive ^218^Po and ^212^Pb aerosols formed by well-characterized set-ups [72]. Alpha- and gamma-counting protocols were used to measure the radioactivity of receiving membrane filters fitted at the inlet and the outlet of the used cast. The same study used a condensation nucleus counter to measure the upstream and downstream NaCl aerosol concentrations passing through the cast model [72].

Shah et al. conducted a clinical study compared to in vitro nasal spray deposition assessment in a sectional nasal cast to provide a proof of concept that deposition of radiolabeled formulations is a valid surrogate for drug distribution in the nose [122]. Gamma-imaging and HPLC were used as quantification techniques to determine the regional distribution of radiolabeled and unlabeled mometasone furoate sprayable nasal suspension formulations, respectively. The deposited dose of radiolabeled (87%) and unradiolabeled (93%) formulations was essentially equal. The regionally depositions of the two were also closely matched within each section. The authors concluded that radiolabeling is an accurate method and reflects drug nasal deposition [122]. Magnetic resonance imaging (MRI) was reported in aerosol lung deposition studies [123] where a contrast agent was added to the aerosolized particles to determine regional deposition. Thorough characterisation in NTBDD studies is important, even qualitative/quantitative assays which can be assessed using functional MRI as elegantly demonstrated by Forbes and co-workers [67]. We look forward to MRI studies of nasal deposition.

## 8. Conclusions

The nasal drug delivery market continues to expand, fuelled by the increased demand for alternative routes of administration for novel drugs and to reach different targets. Therefore, comprehensive nasal deposition analysis of aerosolized formulations is essential to validate the performance and efficiency of nasal products. If deeper nasal penetration beyond the nasal valve can be achieved, this increases the chance for the formulation to reach the olfactory region and achieve direct brain targeting. Such deposition profiles are significantly influenced by the physical properties of formulations, as well as device design and the method of administration.

Despite nasal replicas being widely used as a simple and valuable procedure in deposition evaluation, little information is available about their validation. They have limitations, including using one blocked airway models and applying simplified geometries instead of multi-sectional ones with more precise morphological structures. Research has also often used non-physiological experimental conditions, e.g., steady airflow rather than tidal inhalation patterns, lack of mucus, and no consideration of cilia. Patient administration instructions for the device have also not been considered. Moreover, the analytical methods used in some of the published work for deposition quantification were colorimetry-based, which could lack accuracy, especially for underlying nasal structures. More extensive studies into in vitro nasal deposition using nasal cast models are now required in order to obtain a useful tool to predict aerosol performance and pave the way for formulation and device innovation. More sophisticated and precise nasal replicas that overcome these current shortcomings are needed, together with their use in combination with in vivo studies, to associate deposition profiles with drug response. Hence, there are still numerous opportunities for future research into the optimization of drug deposition for local, systemic, and NTB delivery via the nasal route.

## Figures and Tables

**Table 1:** Preclinical models used for nasal formulations and device characterization.

Model		Type	Advantages	Limitations		Ref
Cell cultures	Primary cells, e.g., human nasal epithelium, porcine respiratory and olfactory cells	In vitro	Close simulation of nasal mucosa Miscellaneous cell composition and diseased cells could be obtained	Limited subculture numbers High risk of contamination Short lifespan	Lack of sufficient reproducibility for powder formulations due to concentrations’ heterogeneity at the cell layers. Deposition method could affect the cell layer integrity	[40,41,42,46]
	Immortalized cells e.g., RPMI 2650, Calu-3		Excellent uniformity Easy to handle and culture	Undesired morphological changes Limited differentiation and the absence of some physiological functionalities		[21,43,44,45]
3D-printed nasal replicas		In vitro	Useful tool to compare different nasal devices, formulations, and inhalation protocols Provides detailed deposition pattern of an aerosol in the nasal cavity Could be combined with other techniques, e.g., NGIs to collect data over the entire respiratory tract	Single-block casts are only qualitativeMonotonousness, where one cast represents one patient’s nasal anatomy, which cannot be generalized to the larger population Lack of important factors for nasal drug delivery, e.g., mucociliary clearance		[53,54,55,56,59,60,61,62]
CPFD		In silico	Quality simulation of total and regional deposition over wide parameter scope, e.g., particle size, velocity, airway geometry, airflow Relatively rapid experiment in comparison to in vitro or in vivo models	Often depends on ideal assumptions, e.g., simplified airway geometries, monodispersed particles, and stable inhalation patterns, which otherwise make numerical simulation challenging Many inhalation devices and aerosol generation process cannot be fully simulated		[51,52]
Excised nasal mucosa from animal or human donor		Ex vivo	Genuine nasal tissue with preserved integrity and permeation properties Same tissue could be utilized for other tests, e.g., histological analysis for formulation safety	Complicated model due to differences between species, e.g., tissue thickness and enzymatic activity Proficient tissue handling is requiredAnalytical interference could occur with other bio-composites		[47,48,49,50]
Animal models, e.g., mice, rats, monkeys		In vivo	Surrogates for humans where extensive preclinical testing can be conducted Pharmacokinetic (C_max_, T_max_, AUC, %DTE *, %DTP **) and pharmacodynamic data are obtained	Inter-species anatomical and physiological variations as well as different inhalation profiles		[16,17,19,20,36]

* Drug targeting efficiency percentage (%DTE); ** Nose-to-brain direct transport percentage (%DTP).

**Table 2:** Particle/droplet size distributions of nasal aerosol and the reported experimental regional depositions.

Particle/Droplet Sizes (µm)	Formulation	Device	Regional Deposition *	Ref.
48.3	Suspension	Four nasal spray pumps VP-7	35.4% anterior, 64.4% turbinates	[64]
60.7		PF-35	37.5% anterior, 62.5% turbinates	
61.6		PF-60	43.4% anterior, 56.5% turbinates	
58.1		PF-80 (20 L/min flow rate)	59.4% anterior	
5.4 for the particulate formulation ** 37.1 aerosol droplets following actuation	Dry Powder	Uni-dose DP™ (25 L/min flow rate)	50–65% was deposited in the nasal vestibule and 30–40% in deeper compartments all together: olfactory, turbinates, and nasopharynx	[66]
37 **	Aqueous Solution	Nasal spray pump SP270+ with 3959-actuator (15 L/min flow rate)	~50% at the nasal vestibule ~18% middle/upper turbinates (including the olfactory)	[67]
1–2	Aqueous Solution	Vibrating orifice aerosol generator (15 L/min flow rate)	4% total and <1% each section	[68]
5.1			16% total and 1–4% each section	
10.3			40% anterior, 12% turbinates, 5% in the olfactory	
14.3			65% anterior, 10% turbinates, 2% in the olfactory	
5–7	Oily Solution	Vibrating orifice aerosol generator (30 L/min flow rate)	25–40% anterior, 8% middle, 0–1% posterior	[71]
8–10			55–70% anterior, 5% middle, 0–1% posterior	
15.7 for the particulate formulation ** 266 aerosol droplets following actuation	Suspension	MAD Nasal^TM^ atomization device	25% vestibule, 42% posterior, 33% nasopharynx	[73]
47.3 for the particulate formulation ** 132.4 aerosol droplets following actuation			22% vestibule, 25% posterior, 52% nasopharynx	

* Regional definitions and cast sections are somewhat subjective for each author. ** Dv_50_.

**Table 3:** Nasal drug delivery devices.

Device	Specifications	Regional Deposition Reported	Ref.
Squeeze bottle	Liquid formulations, distributing relatively large volume, e.g., 80 mL, not recommended for children	Wide and deep distribution in the nasal cavity but poor delivery to the superior and posterior regions	[83]
Spray pumps (metered-dose, single/duo-dose)	Most dominated, liquid, and powder formulations and different systems of pumps were introduced to avoid the use of preservatives	Smaller coverage area than nebulizers with high anterior and lower deposition, not suitable for olfactory delivery	[61,64,83,87,88]
Powered nebulizers/atomizers	Liquid formulations, need compressed gasses/mechanical power/ultrasonics to produce small and low-speed aerosol droplets	Middle and superior meatuses. Mesh-type nebulizers achieved greater dorsal deposition than Jet-type when normal or bidirectional nasal delivery is applied. Deeper deposition could be achieved when using a narrow-tip adaptor. A nitrogen-driven nasal atomizer is under development for N2BDD	[61,85,90,91]
Breath-powered bidirectional technique combined with any nasal devices, e.g., nasal sprays, nebulizers	Liquid and powder formulations, exhalation delivery mechanism that causes velum closure preventing formulation run off into the oral cavity, minimize lung deposition	Throughout the nasal cavity with an improved superior and posterior deposition where the olfactory region is located	[83,85,87,88,90]
